# COVID-19 cases, hospitalizations and deaths in Belgian nursing homes: results of a surveillance conducted between April and December 2020

**DOI:** 10.1186/s13690-022-00794-6

**Published:** 2022-01-29

**Authors:** Eline Vandael, Katrien Latour, Esma Islamaj, Laura Int Panis, Milena Callies, Freek Haarhuis, Kristiaan Proesmans, Brecht Devleesschauwer, Javiera Rebolledo Gonzalez, Alice Hannecart, Romain Mahieu, Louise de Viron, Etienne De Clercq, Anne Kongs, Naïma Hammami, Jean-Marc François, Dominique Dubourg, Sarah Henz, Boudewijn Catry, Sara Dequeker

**Affiliations:** 1 Department of Epidemiology and public health, Sciensano, Brussels, Belgium; 2 Department of Veterinary Public Health and Food Safety, Ghent University, Merelbeke, Belgium; 3 Department of Infectious Disease Prevention and Control, Common Community Commission, Brussels-Capital Region, Brussels, Belgium; 4 Iriscare - Brussels public agency for health and social care, Brussels, Belgium; 5 Department of Welfare, Public Health and Family, Government of Flanders, Brussels, Belgium; 6 Agency for Care and Health, Infection Prevention and Control, Government of Flanders, Brussels, Belgium; 7 Direction de la recherche, de la statistique et de la veille des politiques, Agence pour une Vie de Qualité (AVIQ), Charleroi, Belgium; 8 Ministerium der Deutschsprachigen Gemeinschaft, Eupen, Belgium; 9 Faculty of Medicine, Université libre de Bruxelles (ULB), Brussels, Belgium

**Keywords:** COVID-19, Nursing homes, Surveillance, Epidemiology, Belgium

## Abstract

**Background:**

In Belgium, the first COVID-19 death was reported on 10 March 2020. Nursing home (NH) residents are particularly vulnerable for COVID-19, making it essential to follow-up the spread of COVID-19 in this setting. This manuscript describes the methodology of surveillance and epidemiology of COVID-19 cases, hospitalizations and deaths in Belgian NHs.

**Methods:**

A COVID-19 surveillance in all Belgian NHs (*n* = 1542) was set up by the regional health authorities and Sciensano. Aggregated data on possible/confirmed COVID-19 cases and hospitalizations and case-based data on deaths were reported by NHs at least once a week. The study period covered April–December 2020. Weekly incidence/prevalence data were calculated per 1000 residents or staff members.

**Results:**

This surveillance has been launched within 14 days after the first COVID-19 death in Belgium. Automatic data cleaning was installed using different validation rules. More than 99% of NHs participated at least once, with a median weekly participation rate of 95%. The cumulative incidence of possible/confirmed COVID-19 cases among residents was 206/1000 in the first wave and 367/1000 in the second wave. Most NHs (82%) reported cases in both waves and 74% registered ≥10 possible/confirmed cases among residents at one point in time. In 51% of NHs, at least 10% of staff was absent due to COVID-19 at one point. Between 11 March 2020 and 3 January 2021, 11,329 COVID-19 deaths among NH residents were reported, comprising 57% of all COVID-19 deaths in Belgium in that period.

**Conclusions:**

This surveillance was crucial in mapping COVID-19 in this vulnerable setting and guiding public health interventions, despite limitations of aggregated data and necessary changes in protocol over time. Belgian NHs were severely hit by COVID-19 with many fatal cases. The measure of not allowing visitors, implemented in the beginning of the pandemic, could not avoid the spread of SARS-CoV-2 in the NHs during the first wave. The virus was probably often introduced by staff. Once the virus was introduced, it was difficult to prevent healthcare-associated outbreaks. Although, in contrast to the first wave, personal protective equipment was available in the second wave, again a high number of cases were reported.

**Supplementary Information:**

The online version contains supplementary material available at 10.1186/s13690-022-00794-6.

## Background

The SARS-CoV-2 virus (severe acute respiratory syndrome coronavirus-2) had spread rapidly across the world with almost 93 million confirmed cases in 190 countries and nearly two million deaths at the beginning of 2021 [1]. In Belgium, the first COVID-19 case was reported in February 2020 and on 10 March 2020, the first COVID-19 death was confirmed [2]. As reported in other countries [3, 4], older adults (65 years and older) were the most affected age group, representing 94% of all Belgian COVID-19 deaths between 10 March 2020 and 14 February 2021 [5]. Belgium was ranked among the countries with the highest COVID-19 mortality in the world during its first COVID-19 wave [6, 7]. Based on the cumulative results on the COVID-19 dashboard of the European Centre for Disease Prevention and Control (ECDC) on the 3rd of January 2021, Belgium ranked on the second place among European countries with 1735 COVID-19 deaths per million population. When looking at the number of cases, Belgium stood on the 10th place with 5691 COVID-19 cases per million population [8]. However, differences in reporting practice should be taken into account when comparing countries.

Similar to other European countries, Belgium has an aging population with approximately 19% of the population older than 65 years. Of the latter group, 5.7% is living in nursing homes for elderly (NHs) [9]. Belgium has one of the highest number of NH beds per 1000 population in Europe and tops the chart with the number of people of 85 years and older living in an institution [10].

Globally, the pandemic has been catastrophic for many NHs [11], and the Belgian institutions are no exception. Due to their advanced age and the high prevalence of comorbidities, NH residents are extra vulnerable to communicable diseases, COVID-19 in particular. Moreover, they have frequent close contacts with caregivers and other residents [12, 13], leading to a high risk of widespread transmission and outbreaks of viruses, such as SARS-CoV-2. Staff members and visitors, who daily interchange between the NH and the community, can easily introduce the virus in the NH [14]. Because of the combination of high vulnerability and outbreak-potential, it was essential to follow-up the spread of COVID-19 in these settings.

In March 2020, immediately after the first Belgian COVID-19 cases were reported, surveillance systems were set up, not only to follow-up the COVID-19 situation among the general population, but also among NH residents and the concerned healthcare workers. In May 2020, ECDC recommended the implementation of local and national monitoring systems for COVID-19 in long-term care facilities (LTCFs) such as NHs [15].

The aim of this manuscript is to describe the methodology of the COVID-19 surveillance in Belgian NHs during the study period (April–December 2020) as well as the epidemiology of the reported cases (residents and staff), hospitalizations (residents) and deaths (residents) due to COVID-19 in these NHs prior to the start of the vaccination campaign (5th of January 2021).

## Methods

### Data collection

Since NHs fall under the responsibility of the federated entities, the COVID-19 surveillance in NHs was organized by the regional health authorities (RHA). Each RHA collected the data, analysed them and organised interventions accordingly on a daily basis. The data of all regions were merged in one database and analysed at national level by Sciensano, the Belgian institute for health. More details of the different regional data collection tools and methods can be found in Additional file 1.

All Belgian NHs (*n* = 1542) were invited to participate in the surveillance. Although strongly encouraged and being contacted in case of non-participation by the RHA, the number of NHs that reported data varied. The surveillance started in March 2020 and was initially based on a daily collection of aggregated data reported by NH staff. Since early July, NHs (except for Flanders) were asked to report at least once a week (preferably on Tuesday) in case of zero reporting. Any changes (e.g. new COVID-19 cases, change in the prevalence) had to be reported immediately (see Additional file 1). Collected variables and applied definitions can be found in Tables 1 and 2, respectively.

**Table 1:** *Collected variables in the COVID-19 surveillance in Belgian nursing homes* [16]

Date of registration	
**NH**^**a**^ **characteristics**	- Name - Unique identification number - Postal code - Number of beds - Number of occupied beds (including beds occupied by hospitalized residents and short-stay residents) - Number of staff^b^
**Residents with confirmed or possible COVID-19 infection**	- Number of newly reported confirmed/possible COVID-19 cases since the last reporting - Total number of confirmed/possible COVID-19 cases at the moment of registration
**Hospitalizations of residents due to COVID-19**	- Number of newly reported confirmed/possible COVID-19 cases admitted to a hospital since the last reporting
**COVID-19 deaths among residents**^**c**^	Case-based data of deceased residents - Date of birth - Date of death - Gender - Method of diagnosis (lab-confirmed, computed tomography (CT thorax) confirmed, possible) - Place of death (NH, hospital, other)
**Staff**^**b**^ **with confirmed or possible COVID-19 infections**	- Number of newly reported confirmed/possible COVID-19 cases since the last reporting - Total number of confirmed/possible COVID-19 cases absent from work at the moment of registration (available since 12 May 2020 for all regions)

^a^Nursing homes (NHs) in Belgium can be defined as collective residences in which mainly older people, with different levels of disabilities and independence, are living on a long-term basis (usually until the end of their life). NHs offer a home substitute environment when care can no longer be adequately provided at home or with a short-term admission in a residential institution [17, 18]

^b^All personnel working in the facility, including nursing staff, paramedical staff, animation team, staff concerned with cleaning, maintenance or quality control, and NH managers and their administrative staff, but excluding students and volunteers

^c^More information on the methodology of the COVID-19 death surveillance can be found in Renard et al. [7]

**Table 2:** *Applied definitions in the COVID-19 surveillance in Belgian nursing homes (NHs)* [19]

Confirmed COVID-19 case	
Before 12 May 2020	Person with laboratory confirmation of SARS-CoV-2, irrespective of clinical signs and symptoms.
Since 12 May 2020	Person with laboratory confirmation of SARS-CoV-2 via a molecular test (polymerase chain reaction (PCR) or rapid antigen test), irrespective of clinical signs and symptoms. A symptomatic confirmed case stays a confirmed case until 14 days after the onset of signs/symptoms AND with at least 3 days without fever AND with a significant improvement in respiratory symptoms. An asymptomatic confirmed case stays a confirmed case until 14 days after the positive test. If the resident has a negative laboratory test within this time span, he/she remains a confirmed case.
**Possible COVID-19 case**	
Before 12 May 2020	Person with an acute upper or lower respiratory tract infection not present before OR who deteriorated if the patient shows chronic respiratory symptoms.
Since 12 May 2020	Person - with at least one of the following main signs/symptoms of an acute viral infection: cough, dyspnoea (shortness of breath), thoracic pain, acute anosmia (loss of the sense of smell) or dysgeusia (distortion of the sense of taste) without obvious cause; OR - with at least two of the following signs/symptoms: fever, muscle pain, fatigue, rhinitis, sore throat, headache, anorexia, watery diarrhoea with no obvious cause, acute confusion, sudden fall with no obvious cause; OR - with worsening of chronic respiratory symptoms (chronic obstructive pulmonary disease, asthma, chronic cough,…); OR - who did not have a laboratory test or whose laboratory test is negative, but who is diagnosed with COVID-19 based on a suggestive clinical presentation and a compatible computed tomography (CT) thorax.

### Data analyses

Data until 3 January 2021 (week 53 of 2020) were used for the analyses. Automatic data cleaning was performed using different validation rules. Aberrant values were set to missing in the database. More information on the data cleaning process can be found in Additional file 2.

Data were divided in three periods: the first wave (W1, 30 March 2020 to 21 June 2020, week 14–25; for data on COVID-deaths: starting on 11 March (week 11)), an interwave period (22 June 2020 to 30 August 2020, week 26–35), and the second wave (W2, start date: 31 August 2020, week 36). These waves were defined based on the number of COVID-19 cases in the Belgian general population. W1 started earlier than the end of March, but valid data was only available since the end of March. W2 lasted longer than the end of December but as vaccination campaigns started in January 2021 in Belgian NHs, 3 January 2021 (week 53) was used as end date for the present paper.

Analyses were performed on the number of (new) COVID-19 cases among residents and staff, the number of newly reported hospitalizations related to COVID-19 among residents and the number of COVID-19 deaths among residents. The sum of the number of possible and confirmed COVID-19 cases was used in the analyses, as testing was not yet possible in the beginning of the study period in NHs (reserved for hospitalized patients only). In addition, specific results for confirmed COVID-19 cases only are also shown for the interwave period and W2.

Incidence data were presented as the sum per week of newly reported possible and confirmed cases per 1000 NH residents or staff members. Prevalence data were presented as the total number of reported possible or confirmed cases at the moment of registration per 1000 NH residents or staff members. As the participation rate was overall the highest on Tuesdays (as since 1 July, NHs were conveniently asked in certain regions to report at least once a week on Tuesday), weekly prevalence data were estimated based on data of Tuesday (or Wednesday if Tuesday was a public holiday). Weeks are always displayed from Monday to Sunday. Both crude overall prevalence and incidence rates (total number of (new) cases over the total number of residents or staff in all NHs) and prevalence and incidence rates per NH were calculated.

The number of occupied beds at the moment of reporting was used as denominator for the total number of residents (or number of beds if not available). To have one denominator per NH, the median bed occupation per NH was used for the period of analysis (per week, wave or total study period). For staff, a fixed number at the beginning of the crisis was used (first entry between March–May 2020). To correct for varying participation rates, only NHs that reported data during the concerned time period were taken into account in the denominator. NHs with missing data for a certain variable were excluded in the analyses of that particular variable (both in the numerator and denominator).

Deaths among NH residents that were due to a clinically compatible disease are included in the analysis, regardless of the type of COVID-19 classification (laboratory molecular test, radiologically confirmed case, and by means of clinical criteria) [7]. For hospital deaths between 13 March 2020 and April 2020 information about the residence (whether it concerned a NH resident or not) was not available for Flanders. The number of COVID-19 deaths of NH residents that died in a hospital in this period were therefore estimated using a correction factor. These estimations were excluded in the analyses of age/gender distribution of deceased residents and the distribution of the number of COVID-19 deaths per NH. In the latter analysis, deaths that occurred in hospitals that could not be linked to a specific NH were excluded from the analysis.

Data cleaning and analyses were performed using SAS Enterprise version 7.1. Cumulative numbers, percentages, and medians with interquartile range (IQR) were calculated where appropriate. The number of NHs that reported at least one, two (possible cluster) or ten (possible large outbreak) COVID-19 cases among residents and absenteeism of staff due to COVID-19 (at least 10, 20 or 50% absent) were based on prevalence data. The number of NHs that never declared a COVID-19 case among staff was based on the cumulative incidence (as prevalence data are only available for staff absent from work).

## Results

### COVID-19 infections among residents and staff

Overall, 1529 NHs out of 1542 (99%; median number of beds: 91, IQR: 64–120) participated at least once during the complete study period, with a median weekly participation rate (NHs that participated at least once a week) of 95% (IQR: 93–96%). More details on the participation rate can be found in Additional file 3.

**Fig. 1:**
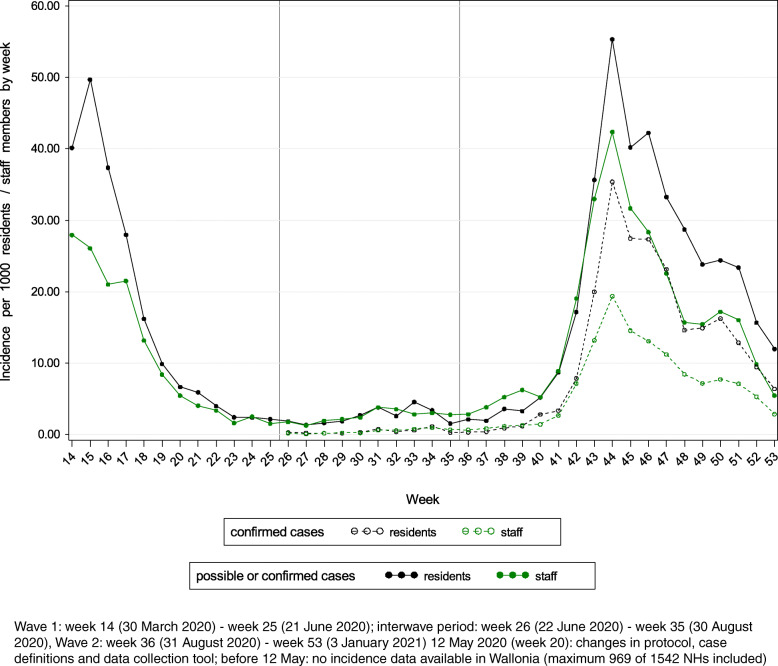
Incidence of COVID-19 cases per 1000 residents/staff members by week

**Fig. 2:**
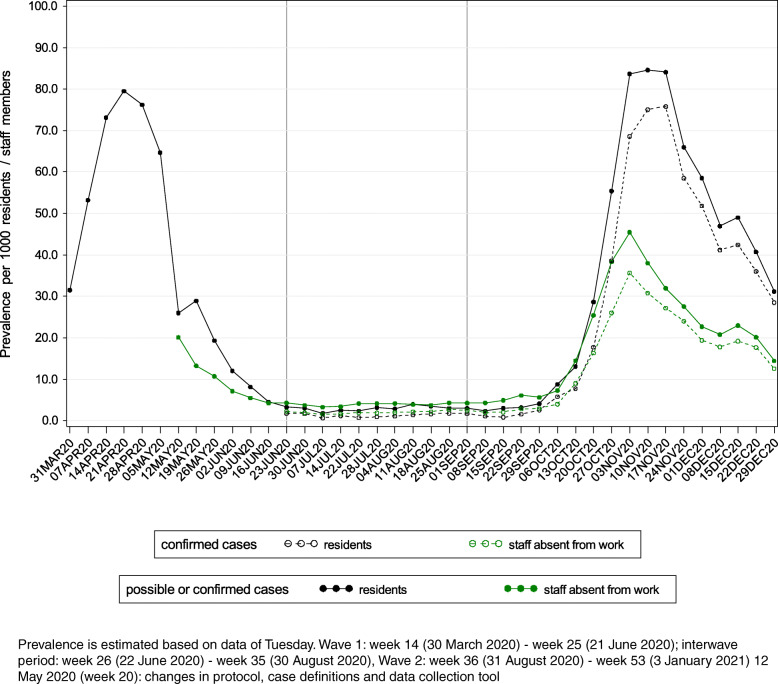
Prevalence of COVID-19 cases per 1000 residents and COVID-19 cases absent from work per 1000 staff members

Figure 1 presents the incidence of COVID-19 cases among NH residents and staff members by week. In Fig. 2, the prevalence of COVID-19 cases is presented for both groups (staff: only cases absent from work). During W1, the peak of the crude incidence per week of possible or confirmed COVID-19 cases among residents (50 cases/1000 residents) was reached in week 15 (week of 6 April 2020). The crude prevalence was the highest on 21 April 2020 (80 cases/1000 residents). For staff, the peak was likely already reached before the data collection had started in all regions. During W2, the peak of the incidence was reached in week 44 (week of 26 October 2020) for both residents (55 cases/1000 residents) and staff members (42 cases/1000 staff members). The highest prevalence was reported on 10 November 2020 for residents (85 cases/1000 residents) and on 3 November 2020 for absent staff members (46 cases/1000 staff members).

The cumulative incidence of possible or confirmed COVID-19 cases at the end of W1 (*n* = 951 NHs, excluding NHs in Wallonia as incidence data were only available from 12 May 2020 onwards in this region) was 206/1000 residents (median per NH: 105/1000 residents, IQR: 30–290/1000 residents) and 139/1000 staff members (median per NH: 81/1000 staff members, IQR: 29–201/1000 staff members). For W2, the cumulative incidence was 367/1000 residents (median per NH: 196/1000 residents, IQR: 51–532/1000 residents) and 284/1000 staff members (median per NH: 194/1000 staff members, IQR: 81–396/1000 staff members). During W2, the cumulative incidence of confirmed cases only was 219/1000 residents (median per NH: 91/1000 residents, IQR: 10–349/1000 residents) and 123/1000 staff members (median per NH: 61/1000 staff members, IQR: 13–165/1000 staff members).

**Fig. 3:**
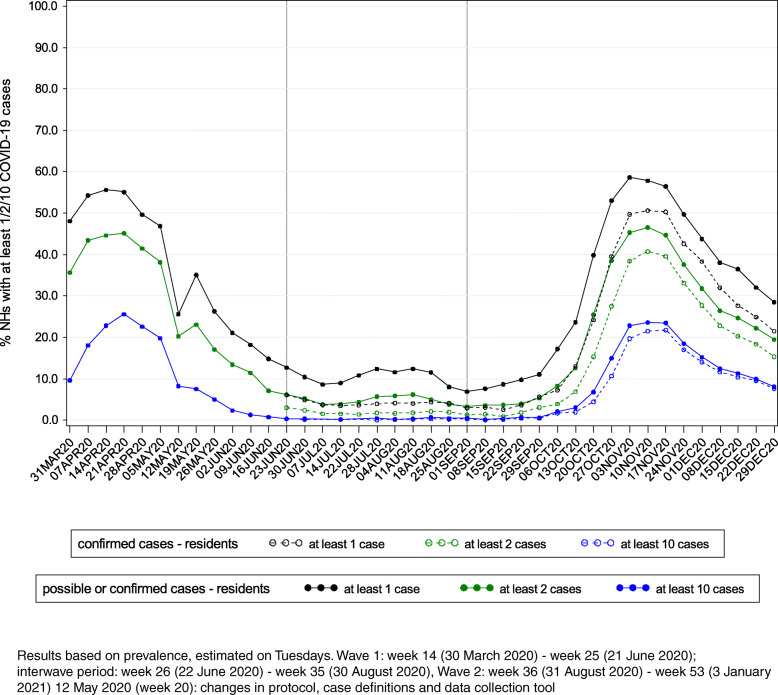
Percentage of participating nursing homes that declared at least 1/2/10 possible/confirmed COVID-19 cases among residents by week

In Fig. 3, the percentage of NHs that declared at least one, two and ten possible or confirmed COVID-19 cases among residents is displayed per week (on Tuesdays). Of all participating NHs, 97, 94 and 74% reported respectively at least one, at least two and at least ten possible or confirmed COVID-19 cases among residents at one point in time (Table 3). Of the 603 NHs that reported at least ten cases during W1, 565 (94%) reported again at least one case and 250 (42%) at least ten cases during W2.

**Table 3:** Number of nursing homes reporting at least 1/2/10 COVID-19 cases among residents and at least 10/20/50% of staff absent due to COVID-19 at one point in time

Nursing homes (*n* = 1542)	Wave 1		Interwave period		Wave 2		All periods		Wave 1 and 2^a^	
	*N*	%	*n*	%	*N*	%	*n*	%	*n*	%
**Residents**										
≥ 1 possible or confirmed case	1355	87.9	699	45.3	1407	91.2	1495	97.0	1269	82.3
≥ 2 possible or confirmed cases	1147	74.4	376	24.4	1277	82.8	1447	93.8	982	63.7
≥ 10 possible or confirmed cases	603	39.1	35	2.3	784	50.8	1140	73.9	250	16.2
≥ 1 confirmed case			298	19.3	1233	80.0				
≥ 2 confirmed cases			134	8.7	1065	68.5				
≥ 10 confirmed cases			19	1.2	681	44.2				
**Staff absent from work**^**b**^										
≥ 10% (possible or confirmed cases)	110	7.1	45	2.9	703	45.6	784	50.8	41	2.7
≥ 20% (possible or confirmed cases)	28	1.8	9	0.6	290	18.8	314	20.4	8	0.5
≥ 50% (possible or confirmed cases)	4	0.3	1	0.1	35	2.3	38	2.5	1	0.1
≥ 10% (confirmed cases)			19	1.2	570	37.0				
≥ 20% (confirmed cases)			4	0.3	212	13.7				
≥ 50% (confirmed cases)			0	0	15	1.0				

^a^During wave 1 as well as wave 2, not cumulative

^b^Data only available since 12 May 2020 (week 20) for the whole country

Results based on prevalence.

Wave 1: week 14 (30 March 2020) - week 25 (21 June 2020); interwave period: week 26 (22 June 2020) - week 35 (30 August 2020), Wave 2: week 36 (31 August 2020) - week 53 (3 January 2021).

Since 12 May, in 784 (51%), 314 (20%) and 38 (2.5%) NHs respectively at least 10, 20 and 50% of staff was absent due to (possible or confirmed) COVID-19 at one point in time. Only 62 NHs (4.0%) never declared a possible or confirmed COVID-19 case among staff during the study period (W1: *n* = 128 (13%); interwave period: *n* = 924 (60%); W2: *n* = 114 (7.4%)). During W1 and W2, respectively 85 (8.8%) and 67 (4.3%) NHs reported at least one possible or confirmed COVID-19 case among residents (spread over the weeks), but no possible or confirmed cases among staff.

### Hospitalizations of nursing home residents due to COVID-19

**Fig. 4:**
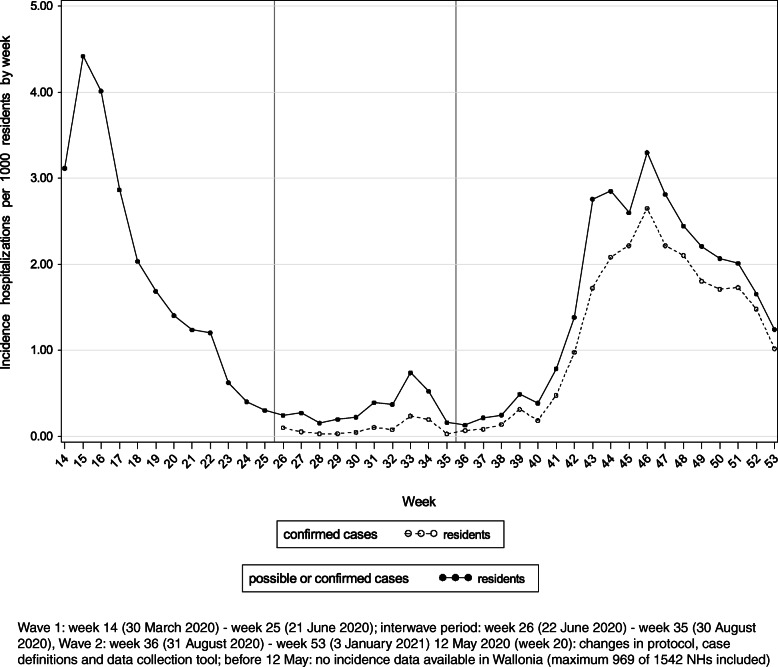
Incidence of hospitalizations due to COVID-19 per 1000 residents by week

Figure 4 displays the incidence of hospitalizations due to possible or confirmed COVID-19 infections per 1000 residents by week. The cumulative incidence of hospitalizations due to (possible or confirmed) COVID-19 infections was 22/1000 residents (median per NH: 8.9/1000 residents, IQR: 0–29/1000 residents) at the end of W1 (*n* = 950 NHs). For W2, this cumulative incidence was 29/1000 residents (median per NH: 11/1000 residents, IQR: 0–39/1000 residents) (*n* = 1518). During W1 and W2, respectively 535 (55%) and 875 (57%) NHs reported at least one hospitalization of a resident due to (possible or confirmed) COVID-19.

### COVID-19 deaths among nursing home residents

During the entire study period (11 March 2020 to 3 January 2021*)*, 11,369 (possible or confirmed) COVID-19 deaths among NH residents were reported (6072 during W1, 150 during the interwave period and 5147 during W2): 76% died in the NHs and 24% died in hospital (22% during W1, 45% during the interwave period and 27% during W2).

**Fig. 5:**
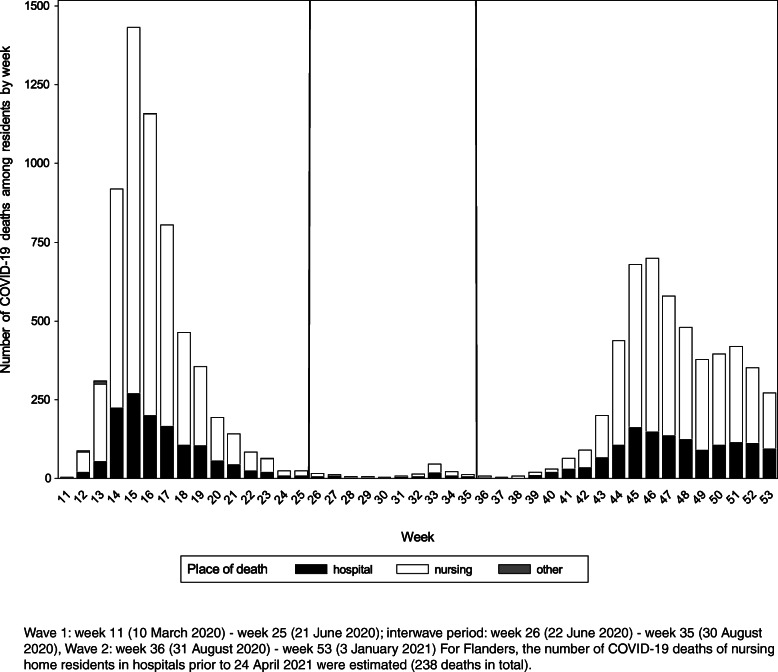
Number of (possible/confirmed) COVID-19 deaths among residents by week

The 11,369 deaths among NH residents represents 57% of all COVID-19 deaths reported in Belgium during this period (*n* = 19,847). The deaths of NH residents accounted for 63% of all COVID-19 deaths in W1, 48% in the interwave period, and 52% in W2. Peaks of COVID-19 deaths among NHs residents were reached in week 15 (*n* = 1433) in W1 and in week 46 (*n* = 703) in W2. Most deaths (73%) occurred in laboratory confirmed cases (55% during W1, 73% during the interwave period, and 95% during W2). The number of COVID-19 deaths among NH residents is presented by week in Fig. 5.

The median number of reported COVID-19 deaths per NH was 7 (IQR 3–13). Overall, 77% of the NHs (*n* = 1188) reported at least 1 COVID-19 death, 68% (*n* = 1044) at least 2 deaths, 50% (*n* = 774) at least 5 deaths and 28% (*n* = 433) at least 10 deaths. The median age of the NH residents that died of COVID-19 during the study period was 88 years (IQR 83–92 years, 61% females).

## Discussion

### Main results

While the applied surveillance was intended in the first place to serve the RHA to manage the crisis and mitigate the epidemic, it allowed us to quantify the COVID-19 pandemic in Belgian NHs prior to the vaccination campaign. Unfortunately and as notified in many countries, Belgian NHs were severely hit by this pandemic. Only a few NHs (3%) never reported any possible or confirmed COVID-19 case among residents. Most NHs (82%) reported cases in both waves. Moreover, absenteeism data show that a considerable number of NHs suffered from staff shortage due to COVID-19 infections among staff members. This is an underestimation of the real absenteeism as other reasons for absence such as chronic conditions like burn-out were not taken into account.

From 12 March 2020 onwards, no visitors were allowed in the NHs for at least 40 days. Although this measure was implemented in the very beginning of the pandemic, it could not avoid the spread of SARS-CoV-2 in the NHs during W1. This suggests that the virus was introduced in this period in many NHs by staff. Asymptomatic cases of staff might have played a role here [20]. Only since 16 April (due to the limited supply of masks in the beginning of the pandemic), the use of surgical masks became recommended for contacts with residents. The initial lack of personal protective equipment (PPE) and infection prevention and control (IPC) training suggests that the majority of cases in W1 were healthcare-associated. Between the end of April and half of May (depending on the regional health authority), a limited number of visitors was allowed again and this remained so until the end of the study period. During the study period, IPC measures of different kind have been implemented at regional and local level (e.g. quarantine measures, cohorting of (possible) cases among residents and staff, use of additional PPE, preventive and rapid testing, ventilation). Each RHA provided detailed instructions for NHs how to deal with COVID-19, but also human resources support initiatives, pro-active calling centres and professional education support. Depending on the available resources and based on the results of the surveillance, outbreak investigations were set up for the largest outbreaks and in NHs with the highest need for local support. Due to important regional and local differences over time it is difficult to assess the impact of the different mitigation measures taken. We can nevertheless reasonably assume that without these measures, W2 would have been much worse [21].

In both waves, the peak in prevalence occurred two weeks after the peak in incidence numbers. On 12 May, it had been clarified in the protocol that a confirmed case remained a case for at least 14 days (or longer depending on symptoms). The cumulative incidence of possible/confirmed cases among residents during W2 was almost twice as high compared to the end of W1, giving the impression that W2 was even worse than W1. One should be careful when comparing these waves. In the beginning of W1, extensive and systematic testing of possible cases in NHs was not possible as free tests were essentially reserved for hospitalized patients. When it became clear that many NHs reported high COVID-19 mortality rates and more tests became available, a massive testing campaign was set up between 8 April and 18 May 2020. This cross-sectional mass testing revealed that about 75% of those that tested positive (residents as well as staff) were asymptomatic at the moment of testing [20]. After this campaign, screening of all residents continued in the context of outbreak investigations, which had an impact on the results. Moreover, over time, NH staff became more familiar with the surveillance, leading possibly to better data registration and quality. Additionally, incidence data were missing for Wallonia in W1, which may have introduced selection bias. All these reasons explain why the results for W2 give a more complete picture and are more robust than those for W1. Nevertheless, despite enough PPE being available in W2 (in contrast with W1), still a high number of COVID-19 cases were reported.

Relatively more residents were hospitalised in W2 as compared to W1. It should be noted that information on residents that refused to be admitted to the hospital and residents that were refused by the hospital due to capacity issues is missing. Moreover, in the beginning of the epidemic, the geriatric society advised openly that residents with COVID-19 should only be referred to a hospital if they would substantially benefit from this hospitalization. Hence, the number of hospitalizations of residents might not be the best indicator to evaluate the severity of the infection. The number of deaths can be considered as a more stable indicator. With 57% of the COVID-19 deaths in 2020 being NH residents, this population (only 1.1% of the total population) was the main group affected. At the beginning of the pandemic, Belgium was acknowledged for its excellent registration including possible COVID-19 deaths in the COVID-19 death statistics, in contrast to many other countries who reported only confirmed cases or even only hospital deaths [7]. The number of deaths of NH residents in W1 was not exceeded in W2 (if counted until 3 January). W2 lasted longer than W1, but peaked less high in terms of deaths. In November 2020, the increase of COVID-19 cases in LTCFs in Belgium and other European countries led to a rapid risk assessment of ECDC. This assessment indicated that several countries were suffering from a second COVID-19 wave in this setting with many fatal cases [11]. Since the NH setting and population can be highly variable across countries, comparing with other countries is difficult.

Since July 2020 (sufficient testing capacity) the 7-day incidence of confirmed COVID-19 cases among NH residents shows a similar (but higher) trend as this incidence in the community. In the community, a sharp increase was observed in week 40 (end of September) [22], whereas in NHs this was observed in week 41. Both the possible introduction by staff (and visitors) and the week delay in the increase of the trend, suggest that the virus was, again in W2, silently introduced from the community. Similar findings have been reported in other countries (UK, Canada, United States) [23–25]. On top, the fact that 51% of the NHs reported a prevalence of at least ten possible/confirmed cases at one moment in time during W2, suggests that once the virus was introduced in the facility, it was still difficult to prevent spread and outbreaks.

### Strengths and limitations

The Belgian COVID-19 surveillance in NHs has been set up very quickly in the context of the pandemic, leveraging the existing experience with infectious disease surveillance in hospitals and LTCFs. Only 14 days after the first COVID-19 death, the surveillance was operational in all regions. The surveillance is part of the national action plan for COVID-19. Several complementary surveillances (e.g. COVID-19 surveillance of confirmed cases in the general population, of cases admitted to Belgian hospitals [26] and of mortality [7]) were put in place and data are linked where possible .

Including possible cases permitted to detect a possible outbreak early and intervene quickly with focussed testing (maximal five tests per NH when an outbreak was suspected in the first months of the epidemic), assistance with PPE, extra staff, psychosocial support and infectious disease management courses. Looking at lab-confirmed cases only would have led to an important underestimation of the incidence/prevalence, especially in the beginning of the COVID-19 crisis. However, it should be taken into account that possible cases might have included respiratory tract infections other than COVID-19 and might have led to an overestimation of the incidence/prevalence of COVID-19.

A high weekly participation rate (median 95%) was reached over the whole study period. Downscaling the registration in some regions from daily to at least once a week in the beginning of July ensured that it remained feasible for all NHs to keep registering data. By collecting also denominator data of residents and staff, relative numbers could be presented making the interpretation over time with varying participation rates easier. A good cooperation between the RHA and Sciensano made it possible to perform analyses on a national level and to visualize the COVID-19 epidemic in Belgian NHs. Since the beginning of the pandemic, detailed weekly reports of the surveillance were made available to the authorities and general public [27]. The COVID-19 surveillance in Belgian NHs has been used as an example in ECDC guidelines [15].

The surveillance also has its limitations. Firstly, as mentioned earlier, the surveillance was set up very quickly in the context of the pandemic by the different RHA, independently from each other. Changes in the protocol, case definitions, data collection tools and variables to be collected were necessary during the first months to respond to the evolving insights in this emerging crisis and to harmonise the data collection. Therefore data of W1 are less robust. Secondly, due to the speed with which this surveillance was set up, there was no time for extensive testing of the different tools. In addition, NH staff were not familiar nor trained to collect surveillance data. Especially in this older population with often vague complaints, it might have been difficult to identify possible cases based on symptoms. Moreover, the surveillance is based on aggregated and not case-based data (except for deaths), which limits the possibilities for data validation (e.g. identification of possible double reported cases, missing cases or delayed reporting), but also for the interpretation of the results. Despite these limitations, it was possible to monitor the epidemic guiding the policy makers in their decisions. However, for more in-depth analyses (e.g. cluster analyses) and to investigate other determinants that played a role (e.g. comorbidities), a case-based registration would be needed. Finally, analyses were limited to the first and second COVID-19 wave and only performed at national level. In future studies, an impact analysis of the vaccination campaign (which started in January 2021) and regional differences could be considered.

## Conclusions

The COVID-19 surveillance in Belgian NHs, essential to guide policy makers, allowed us to map the COVID-19 epidemiology in this setting, despite the limitations of aggregated data and changes in the protocol over time. A high weekly participation rate was achieved throughout the entire study period. The results enabled the RHA to detect a possible outbreak early and to intervene quickly with targeted testing, IPC measures, extra staff and support.

Unfortunately, NHs in Belgium were severely hit by COVID-19, both in W1 and W2, with many fatal cases. W2 lasted longer than W1, but peaked less high in terms of deaths. The measure of not allowing visitors, which was implemented in the very beginning of the pandemic, did not prevent the spread of SARS-CoV-2 in this setting. The virus was probably introduced from the community by staff (or visitors) and once it was introduced, it was difficult to prevent healthcare-associated outbreaks. The majority of NHs reported cases in both waves, indicating that a first outbreak does not guarantee protection for subsequent outbreaks.

## Supplementary Information

**Additional file 1.** Surveillance COVID-19 in Belgian nursing homes (2020), overview data collection in the different regions

**Additional file 2.** Surveillance COVID-19 in Belgian nursing homes (2020), summary of the validation rules for data cleaning

**Additional file 3. **Weekly participation rate of all Belgian nursing homes (NHs, *n*=1542) per period

## Data Availability

The datasets used and/or analysed during the current study are available from the corresponding author on reasonable request.

## Abbreviations

CT: computed tomography
ECDC: European Centre for Disease Prevention and Control
IPC: infection prevention and control
IQR: interquartile range
LTCF: long-term care facility
NH: nursing home
PCR: polymerase chain reaction
PPE: personal protective equipment
RHA: regional health authorities
SARS-CoV-2 virus: severe acute respiratory syndrome coronavirus-2
W1: first wave
W2: second wave
