# Supplementary material for: COVID-19 cases, hospitalizations and deaths in Belgian nursing homes: results of a surveillance conducted between April and December 2020
Source: Arch Public Health. 2022 Jan 29;80:45. doi: 10.1186/s13690-022-00794-6 (PMC8799977; doi:10.1186/s13690-022-00794-6)
Supplement: Supplementary file 1 — Additional file 1. Surveillance COVID-19 in Belgian nursing homes (2020), overview data collection in the different regions [file 13690_2022_794_MOESM1_ESM.docx]

***Additional file 1: Surveillance COVID-19 in Belgian nursing homes (2020), overview data collection in the different regions***

| **Region** | **Regional health authority** | **Number of nursing homes** | **Start data collection** | **Data collection tool** | **Methodology** | **Link to more information** |
| --- | --- | --- | --- | --- | --- | --- |
| Flanders | Agentschap Zorg en Gezondheid (AZG) | 814 | 18/03/2020 | E-loket  (daily transfer of the data to Sciensano) | Throughout 2020: daily registration  From 3 June 2020 to 9 November 2020: reporting only requested on weekdays, not during the weekend  Participation was obligatory. | https://www.zorg-en-gezondheid.be/cijfers-covid-19 |
| Wallonia | Agence wallonne pour une vie de qualité (AViQ) | 573^a^ | 20/03/2020 | Prisma (daily transfer of the data to Sciensano)^c^ | 20 March - 30 June 2020: daily registration  Since 1 July 2020: nursing homes complete the full questionnaire only once a week on Tuesday in case of no changes in the data (no daily confirmation on weekdays is asked). Any changes (e.g. new COVID-19 cases, change in the prevalence) have to be reported immediately. | https://covid.aviq.be/fr/accueil-professionnels |
| Brussels | Commission communautaire commune de Bruxelles Capitale (COCOM) | 147^b^ | 17/3/2020: start collection of COVID-19 deaths  26/03/2020: start surveillance of cases and deaths | Limesurvey set up by Sciensano | 20 March - 30 June 2020: daily registration  Since 1 July 2020: nursing homes complete the questionnaire once a week on Tuesday, in case they have no changes in the data (daily confirmation on weekdays if there were changes or not since the last reporting). Any changes (e.g. new COVID-19 cases, change in the prevalence) have to be reported immediately. | https://www.sciensano.be/sites/ default/files/protocol_covid-19_ surveillance_in_residential_ institutions_20200701_version_3.3.pdf |
| German speaking community | Deutschsprachige Gemeinschaft | 8 | 28/03/2020 | Limesurvey set up by Sciensano | 20 March - 30 June 2020: daily registration  Since 1 July 2020: nursing homes complete the questionnaire once a week on Tuesday, in case they have no changes in the data (daily confirmation on weekdays if there were changes or not since the last reporting). Any changes (e.g. new COVID-19 cases, change in the prevalence) have to be reported immediately. | https://www.sciensano.be/sites/ default/files/protocol_covid-19_ surveillance_in_residential_ institutions_20200701_version_3.3.pdf |

^a^ excluding the nursing homes in the German speaking community

^b^ including 8 nursing homes that fall under the authority of AZG

^c^ 12 May 2020: changes in data collection tool with an impact on the data quality of 12-13 May
