# Supplementary material for: COVID-19 cases, hospitalizations and deaths in Belgian nursing homes: results of a surveillance conducted between April and December 2020
Source: Arch Public Health. 2022 Jan 29;80:45. doi: 10.1186/s13690-022-00794-6 (PMC8799977; doi:10.1186/s13690-022-00794-6)
Supplement: Supplementary file 2 — Additional file 2. Surveillance COVID-19 in Belgian nursing homes (2020), summary of the validation rules for data cleaning [file 13690_2022_794_MOESM2_ESM.docx]

***Additional file 2: Surveillance COVID-19 in Belgian nursing homes (2020), summary of the validation rules for data cleaning***

| Detected errors (in the registrations per day and per nursing home) | How handled in the data cleaning |
| --- | --- |
| Number of COVID-19 cases among residents > total number of residents | Number of COVID-19 cases among residents set to missing for that day/registration |
| Number of hospitalizations due to COVID-19 among residents > total number of residents | Number of COVID-19 hospitalizations due to COVID-19 among residents among residents set to missing for that day/registration |
| Number of COVID-19 cases among staff > total number of staff | Number of COVID-19 cases among staff set to missing for that day/registration |
| Total number of residents / total number of staff >6  Total number of staff / total number of residents >6 | Total number of staff set to missing |
| Number of newly reported COVID-19 cases among residents (incidence) > total number of reported COVID-19 cases among residents (prevalence) | Both variables set to missing |
| Number of newly reported hospitalizations due to COVID-19 among residents (incidence) > total number of reported COVID-19 cases among residents (prevalence) | Number of newly reported hospitalizations set to missing |
| Number of newly reported COVID-19 cases among staff (incidence) > total number of reported COVID-19 cases among staff (prevalence) | Both variables set to missing |
| Total number of residents (current bed occupation) is missing | Replaced by the first number of residents that was filled in since the start of the surveillance, if also missing replaced by the total number of beds |
| Reported date < start date of the surveillance | Regional health authority contacted to receive the corrected data |
| Errors or missing data in the reported COVID-19 deaths | Regional health authority contacted to receive the corrected data; More information in Renard et al. [7] |
