# Supplementary material for: COVID-19 cases, hospitalizations and deaths in Belgian nursing homes: results of a surveillance conducted between April and December 2020
Source: Arch Public Health. 2022 Jan 29;80:45. doi: 10.1186/s13690-022-00794-6 (PMC8799977; doi:10.1186/s13690-022-00794-6)
Supplement: Supplementary file 3 — Additional file 3. Weekly participation rate of all Belgian nursing homes (NHs, n=1542) per period [file 13690_2022_794_MOESM3_ESM.docx]

***Additional file 3: Weekly participation rate of all Belgian nursing homes (NHs, n=1542) per period***

|  | Wave 1 | Interwave period | Wave 2 | All periods |
| --- | --- | --- | --- | --- |
| Number of NHs that participated at least once (%) | 1521  (98.6%) | 1508  (97.8%) | 1519  (98.5%) | 1529  (99.2%) |
| Median weekly participation rate (IQR) | 95.8%  (95.1-96.9%) | 89.7%  (89.4-92.7%) | 95.8%  (94.2-96.1%) | 95.0%  (93.3-96.1%) |
| Maximum weekly participation rate (week in which reached) | 97.0%  (week 18) | 94.9%  (week 35) | 96.8%  (week 50) | 97.0%  (week 18) |
| Minimum weekly participation rate (week in which reached) | 93.6%  (week 21) | 88.5%  (week 30) | 92.5%  (week 39) | 88.5%  (week 30) |

Wave 1: week 14 (30 March 2020) - week 25 (21 June 2020); interwave period: week 26 (22 June 2020) - week 35 (30 August 2020), Wave 2: week 36 (31 August 2020) - week 53 (3 January 2021)

Weekly participation rate: NHs that participated at least once a week
